# TLR7 deficiency enhances inflammation in the URT but reduces LRT immunity following influenza A infection

**DOI:** 10.1038/s41598-025-04154-6

**Published:** 2025-05-29

**Authors:** Mark A. Miles, Sahan Jayawardena, Stella Liong, Felicia Liong, Gemma S. Trollope, John J. O’Leary, Doug A. Brooks, Stavros Selemidis

**Affiliations:** 1 Centre for Respiratory Science and Health, School of Health and Biomedical Sciences, RMIT University, Bundoora, VIC Australia; 2 Discipline of Histopathology, School of Medicine, Trinity Translational Medicine Institute (TTMI), Trinity College Dublin, Dublin, Ireland; 3 Sir Patrick Dun’s Laboratory, Central Pathology Laboratory, St James’s Hospital, Dublin, Ireland; 4 Clinical and Health Sciences, University of South Australia, Adelaide, SA Australia

**Keywords:** Influenza A, Toll-like receptor 7, Inflammation, Upper respiratory tract, Immunology, Infectious diseases, Inflammation

## Abstract

Immune responses in the upper respiratory tract (URT) following influenza A virus (IAV) infection can influence disease severity, and subsequently inflammation and lung tissue damage in the lower respiratory tract (LRT). This study investigated the role of toll-like receptor 7 (TLR7), a key pattern recognition receptor that senses viral RNA and triggers antiviral and proinflammatory signaling to activate immune responses, in specifically shaping URT and LRT immune responses to IAV infection. Wild type C57Bl/6 and TLR7 knockout (TLR7 KO) mice were infected with the H3N2 IAV strain Hk-X31, and key immune responses in the nasal tissue (URT) and lower airways and lung tissue (LRT) measured after acute infection. We found reduced body weight loss, and increased type II/III interferons and proinflammatory cytokines in the URT of TLR7 KO mice; while LRT inflammation was reduced. TLR7 was essential for activating immune responses in the LRT but played a more selective role in the URT, primarily influencing monocytes, pDCs and B cells. Our data suggest that TLR7 plays a critical role in the transition of inflammation from the URT to the LRT during IAV infection, making it a promising therapeutic target to modulate disease severity.

**Supplementary Information:**

The online version contains supplementary material available at 10.1038/s41598-025-04154-6.

## Introduction

Influenza A viruses (IAV) continue to pose a significant global public health threat, contributing to an estimated 5 million cases of severe respiratory illness annually^1^. Infections confined to the upper respiratory tract (URT) are generally mild and self-limiting, with minimal impact on overall health. However, when the disease progresses to the lower respiratory tract (LRT), it can impair respiratory function, potentially leading to severe complications involving hospitalization or death^2^. This disease progression occurs when the virus breaches the mucosal defenses of the URT and triggers an intense LRT inflammatory “cytokine storm”. The immune profiles in the LRT also differ between mild and severe disease, with impaired Th1 and Th17 adaptive responses and poorer viral clearance present in more severe infections^3,4^. Th1 and Th17 cells are subsets of T helper cells that play a key role in the immune response to viral infections. Therefore, a robust antiviral mucosal immune response in the nasal passages plays a critical role in limiting viral spread to the LRT, thereby mitigating the risk of severe disease and complications^5,6^.

The nasal epithelium serves as the primary site of initial exposure to IAV, where the virus readily replicates. In response to IAV, epithelial cells produce type I and III interferons (IFNs) as part of the innate antiviral defenses to limit viral replication and prevent spread^7^. Type III IFN, in particular, plays a key role in restricting the spread of IAV from the URT to the lungs, thus preventing more severe LRT disease involvement^8^. While type I IFNs are effective in limiting viral replication, they also exert additional immunostimulatory effects, including immune cell recruitment and the activation of proinflammatory pathways^9,10^. These secondary effects, while crucial for mounting a broader immune response, can also contribute to excessive inflammation, particularly in the LRT. Epithelial cell death and the consequent breakdown of the epithelial barrier can facilitate viral spread by allowing the virus to access both resident and infiltrating immune cells^11,12^. In particular, the direct infection or activation of alveolar macrophages by damage-associated molecular patterns can exacerbate pulmonary inflammation and contribute to the severity of LRT disease^13^. In the LRT, heightened proinflammatory responses driven by innate immune cells, such as neutrophils and monocytes, along with activated cytotoxic CD8 + T cells, are strongly associated with severe infection and worse clinical outcomes^14–17^. Moreover, elevated cytokine production in the lung can impair the function of virus-specific T cells, natural killer (NK) cells, and antibodies, further contributing to the poor outcomes in severe IAV cases^18^.

Aside from the protective antiviral effects that occur in the URT, dominant proinflammatory signatures in the nasal compartment can influence the subsequent immune dynamics in the LRT. For instance, an enhanced monocyte profile, characterized by elevated levels of MCP-3 and IFNα in nasal lavages, can predict the progression to severe IAV disease. Similarly, increased neutrophilic IL1B, IL-6, and CXCL8 responses in the nasopharyngeal aspirates of IAV infected individuals have been associated with heightened disease severity^17^. Furthermore, pre-existing neutrophilic inflammation in the nasal passages can suppress IL-17-mediated immunity, and can exacerbate pulmonary disease by promoting excessive recruitment of CD8 + T cells to the lungs during respiratory syncytial virus (RSV) infection^19^. Overall, these findings suggest that a dominant proinflammatory response in the URT at the onset of infection may impair early antiviral responses and trigger excessive downstream immune activation in the LRT, potentially driving severe disease progression.

Host immune responses to IAV infection are primarily triggered by the activation of pattern recognition receptors (PRRs), which detect viral components and initiate signaling pathways that drive antiviral and inflammatory responses. Toll-like receptors (TLRs)-7 and - 8, located in the endosome, recognize viral single-stranded RNA (ssRNA) and activate the adaptor protein MyD88. This, in turn, stimulates the transcription of type I IFNs via interferon regulatory factors (IRFs) and proinflammatory cytokines via the NF-κB pathway, helping to establish both innate and adaptive host defenses against the virus. Sensing of viral double stranded RNA (dsRNA) intermediates by other PRRs like TLR3 and cytosolic RIG-I-like receptors (RLRs) activate similar pathways to reinforce antiviral responses^20^. Emerging evidence suggests that dysregulation or hyperactivation of TLR7 may contribute to immunopathology in the lungs. Specifically, excessive activation of TLR7 has been linked to increased host-mediated lung damage during infection with ssRNA viruses including IAV, SARS-CoV-2 and RSV^21–24^. One potential mechanism for this is the hyperactivation of pulmonary macrophages, which express high levels of TLR7 and can adopt a proinflammatory phenotype in response to activation. This exacerbates tissue damage and promotes inflammation, rather than facilitating efficient viral clearance^25^. This shift toward a maladaptive immune response suggests that, although TLR7 plays a crucial role in detecting IAV and initiating antiviral immunity, it can also contribute to the pathogenesis of LRT disease.

Unlike other PRRs, TLR7 responses in the URT during IAV infection are less well understood. TLR7 expression in nasal epithelial cells is relatively low, and its activation capacity in these cells appears limited^26–28^. However, human nasal biopsies from healthy individuals show higher TLR7 expression compared to in vitro cell line models, suggesting that non-epithelial or immune cells in the URT may have the capacity to initiate these TLR7 responses^26^. Notably, when these nasal biopsies were treated with a TLR7 agonist, there was a marked induction of proinflammatory cytokine expression^26^, providing evidence that TLR7 can be activated in the URT. Despite these findings, the precise role of TLR7 in driving inflammation in the URT following IAV infection remains unclear.

Given the association of TLR7 hyperactivation with excessive inflammation and immunopathology in the lungs, we aimed to determine whether TLR7-dependent responses in the URT following IAV infection were linked to subsequent inflammation in the LRT. We found that TLR7 expression and stimulatory capacity were greater in the LRT compared to the URT. Innate and adaptive immune responses to IAV infection were largely compromised in the LRT of TLR7 knockout (KO) mice while monocyte, plasmacytoid dendritic cells (pDC) and B cell responses were selectively impaired in the URT. Furthermore, inflammatory cytokines and type II/III IFNs were elevated in the nasal tissue of TLR7 KO mice compared to wild type (WT) mice, which displayed a higher type I IFN response. These findings highlight the importance of understanding immune dynamics in both the URT and LRT for the development of targeted therapeutic strategies to reduce IAV-induced disease severity.

## Results

### TLR7 expression and function are highest in immune cells of the LRT

**Fig. 1:**
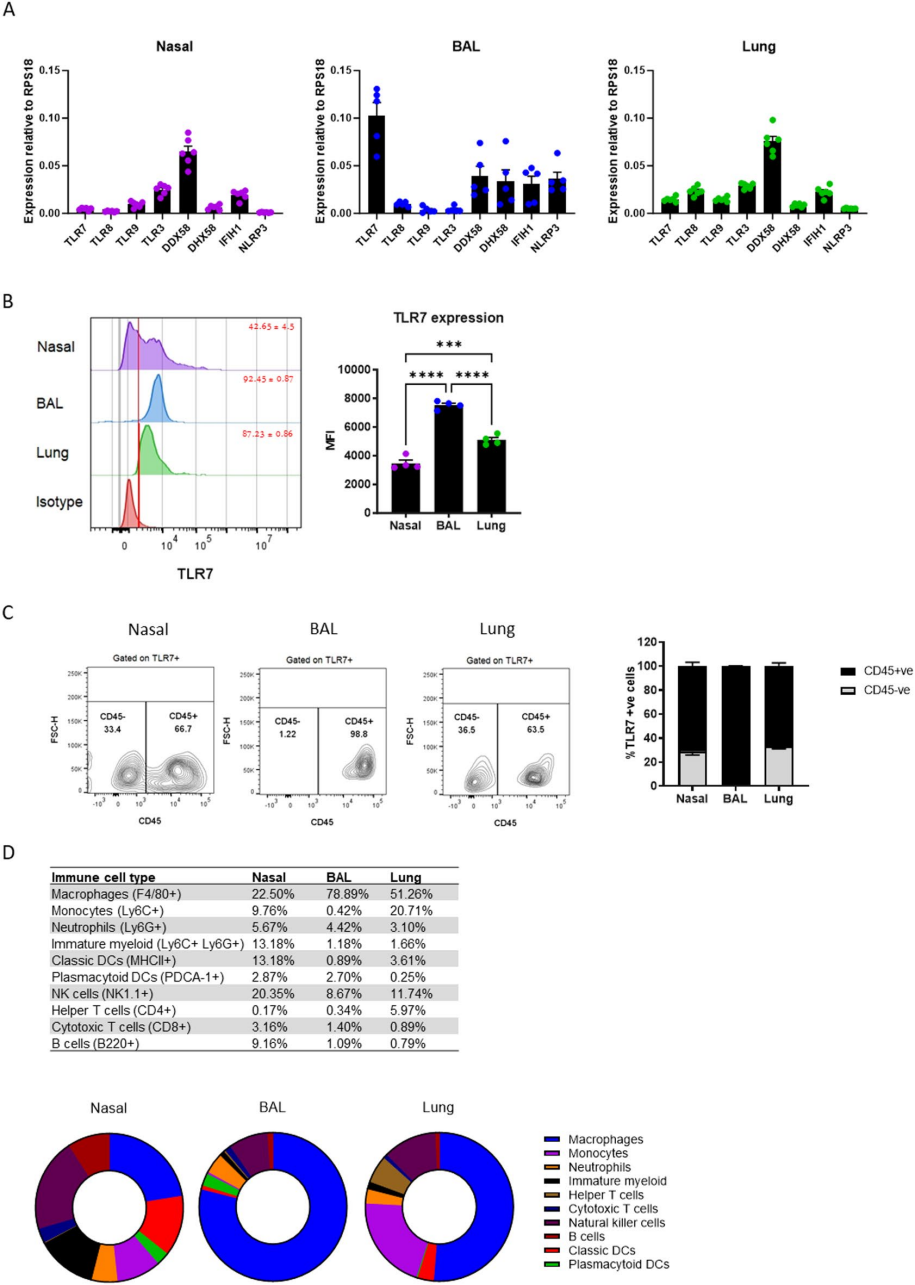
Mapping of TLR7 expression across URT and LRT. Nasal, BAL and lung tissues from uninfected WT C57Bl/6 mice were analyzed. (**A**) Gene expression of pattern recognition receptors was measured by RT-qPCR and presented relative to RPS18 housekeeping (2^−ΔCt^). (**B**) Intracellular staining of TLR7 was determined by flow cytometry with positively stained cells gated above unstained isotype controls. The mean fluorescence intensity (MFI) for each tissue is also presented. The proportion of TLR7 expressing (TLR7+) cells staining for (**C**) CD45 or (**D**) other surface markers (gated on CD45 + cells) to identify immune cell types was determined. Data is expressed as mean ± SEM, *n* = 4–5 biological replicates. Statistical analysis was conducted using one-way ANOVA test followed by Tukey’s post hoc test for multiple comparisons (***p < 0.001, ****p < 0.0001).

To investigate the functional expression of TLR7 in different regions of the respiratory tract, we compared its basal expression levels in uninfected nasal tissue (representing the URT), cells enumerated from bronchoalveolar lavage (BAL) and lung tissues (representing the airways and lung tissue of the LRT, respectively), alongside other PRRs known to play roles in viral sensing. *TLR7* transcripts were detected in all samples, but with high expression found in the BAL, and lower expression in the lung and nasal tissues (Fig. 1A). In contrast, the expression of dsRNA sensors *DDX58/RIGI* was highest in nasal tissue, followed by *TLR3* and *IFIH1/MDA5*. A similar expression pattern was observed in the lung, although *TLR8* and *TLR9* expression levels were comparable to those of *TLR7*. BAL cells also expressed various *RLRs* and *NLRP3*, although these were expressed at approximately half the levels of *TLR7*. Minimal expression of other endosomal *TLRs* was detected in the BAL.

We next confirmed the intracellular expression of TLR7 at the protein level by flow cytometry. Consistent with gene expression data, intracellular protein levels of TLR7 were highest in the BAL, followed by the lung then nasal tissues (Fig. 1B). Approximately 65% of TLR7-expressing cells in the nasal and lung tissues were immune cells, compared to more than 98% in the BAL (Fig. 1C). Further analysis revealed the specific immune cell types expressing TLR7 in these samples (Fig. 1D). In nasal tissue, TLR7 was expressed by macrophages and NK cells, with lower expression in monocytes, Ly6C + Ly6G + myeloid cells, classic DCs and B cells. In the BAL, approximately 80% of TLR7-expressing cells were macrophages, with fewer being NK cells and neutrophils. In lung tissue, about half of the TLR7 + cells were macrophages with monocytes and NK cells making up approximately one third of the TLR7-expressing population.

**Fig. 2:**
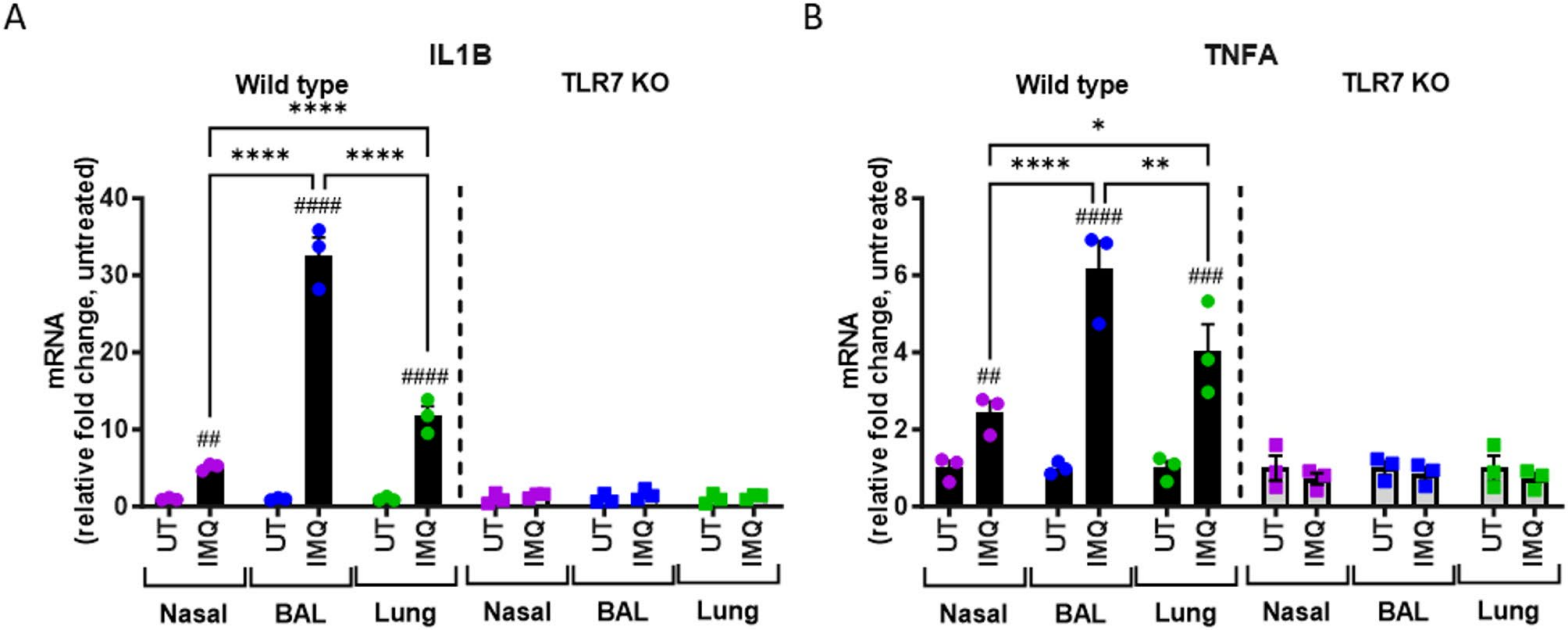
TLR7 stimulation promotes upregulation of proinflammatory cytokines predominantly in the LRT. Nasal, BAL and lung cell suspensions from WT C57Bl/6 or TLR7 KO mice were treated with 1 µg/ml imiquimod (IMQ) or left untreated (UT) for 18 h. Gene expression of (**A**) *IL1B* and (**B**) *TNFA* was measured by RT-qPCR and presented relative to RPS18 housekeeping as a fold change above UT controls. Data is expressed as mean ± SEM, *n* = 3 technical replicates from 3 biological replicates for each strain. Statistical analysis was conducted using two-way ANOVA test followed by Tukey’s post hoc test for multiple comparisons (*p < 0.05, **p < 0.01, ****p < 0.0001 for comparison between tissues; ##*p* < 0.01, ###*p* < 0.001, ####*p* < 0.0001 for comparison with each UT control).

To determine the capacity for TLR7 responsiveness in each tissue, we generated cell suspensions from WT and TLR7 KO mice and stimulated them with the TLR7 agonist imiquimod (IMQ). Upon stimulation, the transcription of *IL1B* and *TNFA* was upregulated in all WT tissues, but not in TLR7-deficient cells, confirming that IMQ-induced proinflammatory gene activation is TLR7 dependent (Fig. 2A, B). Consistent with TLR7 expression levels, the greatest inflammatory gene upregulation in response to IMQ occurred in the BAL, followed by the lung, and then the nasal tissue.

These analyses demonstrate that TLR7 is predominantly expressed in immune cells within both the URT and LRT, with macrophages constituting the most frequent cell type in the absence of infection. Moreover, the expression and activation of TLR7 differ between the upper and lower compartments, with TLR7-dependent inflammation being most pronounced in the BAL.

### TLR7 deficiency alters PRR expression predominantly in the URT following IAV infection

To interrogate whether loss of TLR7 impacted PRR expression in the URT and LRT following IAV infection, we intranasally inoculated WT and TLR7 KO mice with X31 virus (H3N2 strain) and analysed tissue after 6 days. Acute infection resulted in body weight loss in both WT and TLR7 KO mice, although WT mice lost significantly more weight than TLR7 KO mice (Supplementary Fig. 1), suggesting that the infection was more severe in TLR7-expressing mice.

**Fig. 3:**
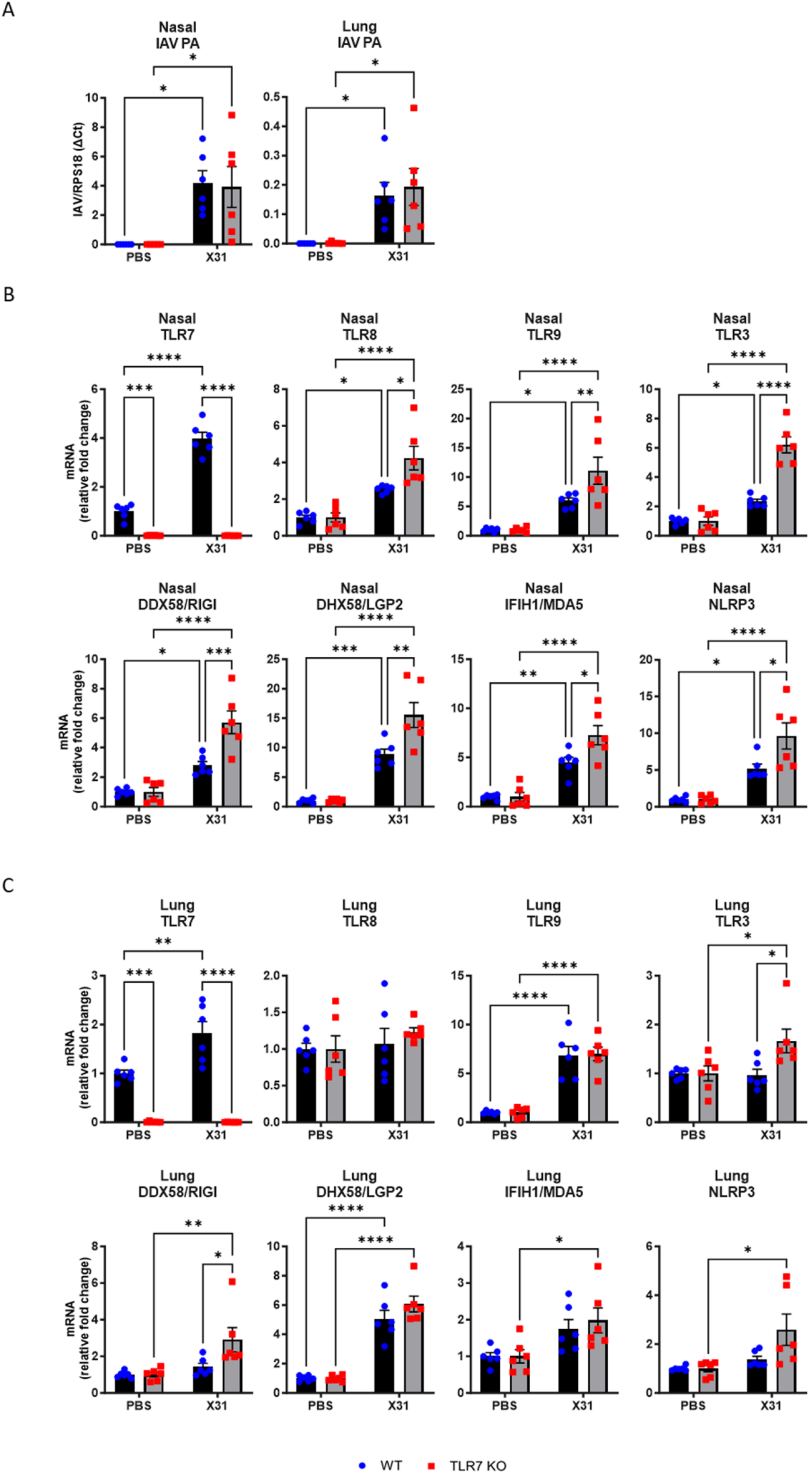
TLR7 KO mice exhibit increased PRR expression in URT following X31 infection. WT C57Bl/6 or TLR7 KO mice were infected with Hk-X31 (10^4^ PFUs) or PBS (control). Nasal and lung tissues were isolated after 6 days and gene expression analysed by RT-qPCR. (**A**) Influenza A virus polymerase transcripts are presented relative to RPS18 housekeeping (2^−ΔCt^). Expression of various PRRs in the (**B**) nasal and (**C**) lung tissues are presented relative to RPS18 housekeeping as a fold change above PBS controls. Data is expressed as mean ± SEM, *n* = 3 technical replicates from 6 biological replicates for each strain. Statistical analysis was conducted using two-way ANOVA test followed by Tukey’s post hoc test for multiple comparisons (*p < 0.05, **p < 0.01, ***p < 0.001, ****p < 0.0001).

TLR7 deficiency did not affect viral transcript levels in either nasal or lung tissues (Fig. 3A). In the nasal tissue, the expression of endosomal TLRs, cytosolic RLRs and NLRP3 increased with infection in both genotypes, but levels (with the exception of *TLR7*) were higher in TLR7 KO mice (Fig. 3B). In contrast, only TLR7, TLR9, and DHX58 were elevated in the lungs of WT mice, and TLR7 deficiency did not alter this response (Fig. 3C). While lung expression of *TLR3*, *DDX58* and *NLRP3* remained unchanged in WT mice following infection, it was significantly higher in TLR7 KO mice. This differential expression of PRR in the nasal and lung tissues was dependent on TLR7, suggesting that immune responses in both the upper and lower respiratory compartments were likely to be altered as well.

### TLR7 deficiency upon IAV infection largely alters the immune profile in the LRT but only affects monocytes, pDCs and B cells in the URT

**Fig. 4:**
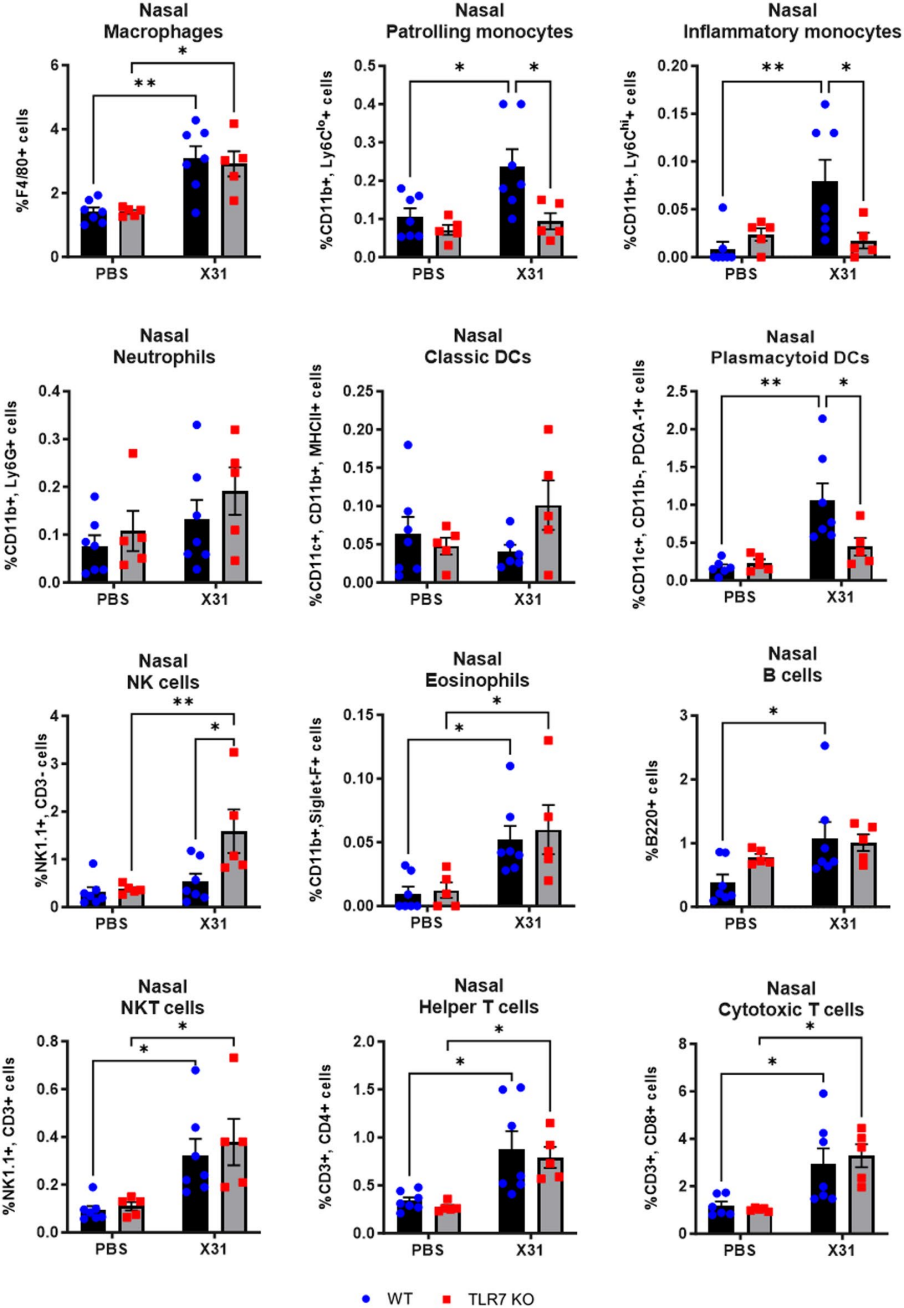
TLR7 KO mice exhibit reduced monocyte, pDC and B cell recruitment in the nasal tissue following X31 infection. WT C57Bl/6 or TLR7 KO mice were infected with Hk-X31 (10^4^ PFUs) or PBS (control). Immune cell populations in the nasal tissue after 6 days were determined by flow cytometry. Data is expressed as mean ± SEM, *n* = 5–7 biological replicates. Statistical analysis was conducted using two-way ANOVA test followed by Tukey’s post hoc test for multiple comparisons (*p < 0.05, **p < 0.01).

**Fig. 5:**
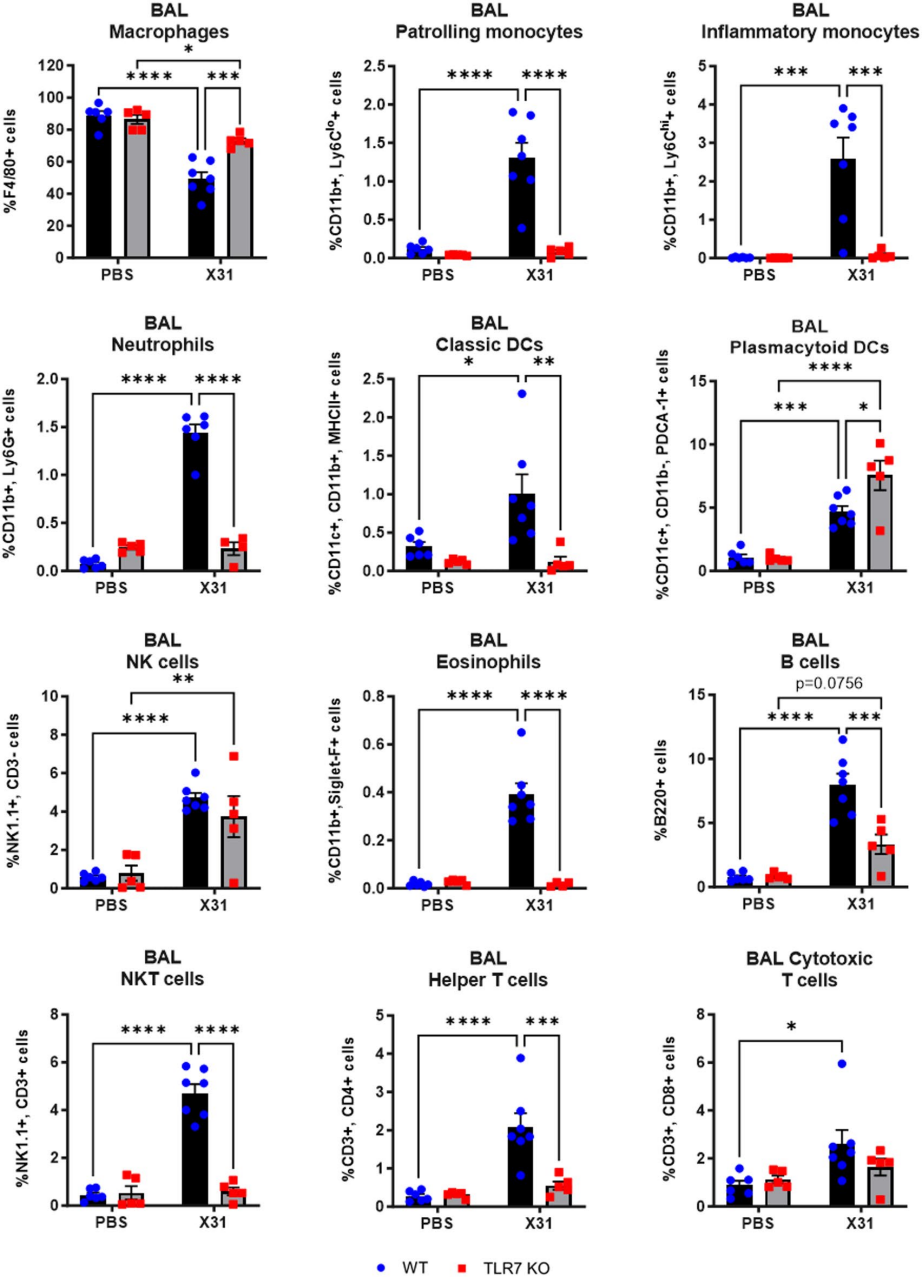
TLR7 KO mice exhibit reduced innate and adaptive immune cell recruitment in the lower airways following X31 infection. WT C57Bl/6 or TLR7 KO mice were infected with Hk-X31 (10^4^ PFUs) or PBS (control). Immune cell populations in the BAL after 6 days were determined by flow cytometry. Data is expressed as mean ± SEM, *n* = 5–7 biological replicates. Statistical analysis was conducted using two-way ANOVA test followed by Tukey’s post hoc test for multiple comparisons (*p < 0.05, **p < 0.01, ***p < 0.001, ****p < 0.0001).

**Fig. 6:**
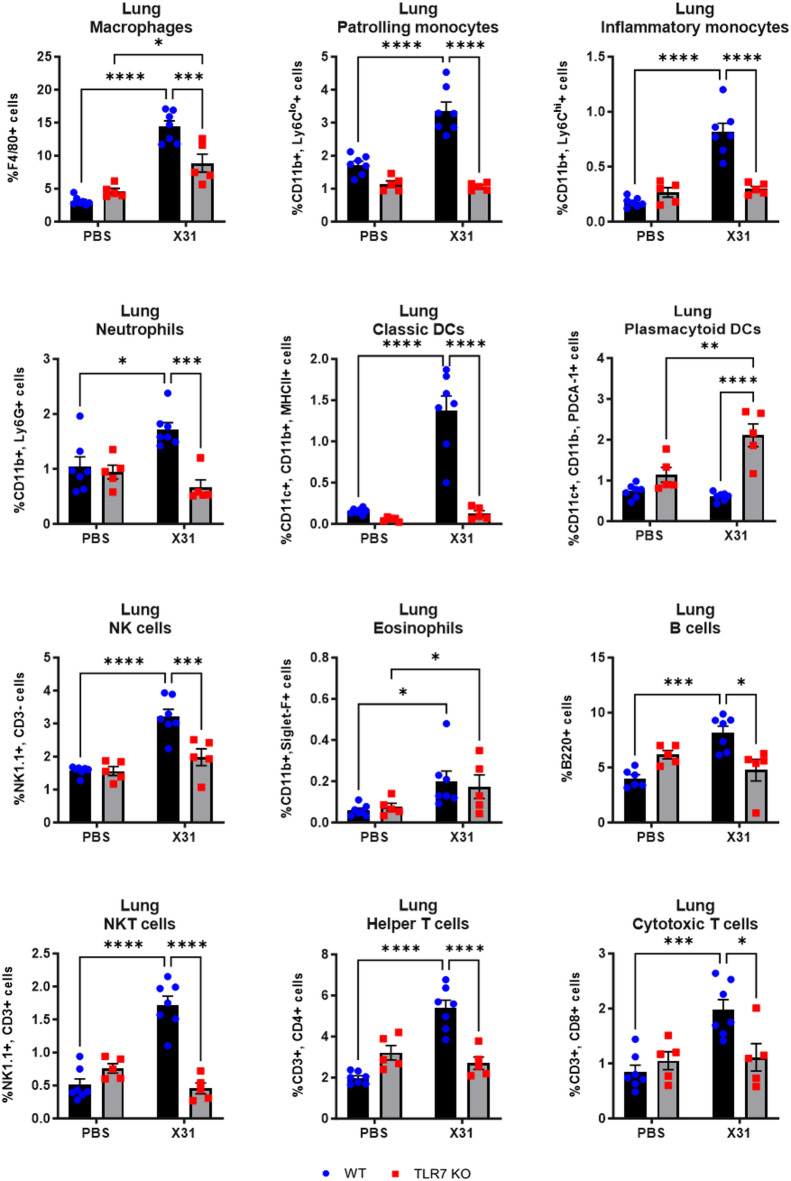
TLR7 KO mice exhibit reduced innate and adaptive immune cell recruitment in the lungs following X31 infection. WT C57Bl/6 or TLR7 KO mice were infected with Hk-X31 (10^4^ PFUs) or PBS (control). Immune cell populations in the lungs after 6 days were determined by flow cytometry. Data is expressed as mean ± SEM, *n* = 5–7. Statistical analysis was conducted using two-way ANOVA test followed by Tukey’s post hoc test for multiple comparisons (*p < 0.05, **p < 0.01, ***p < 0.001, ****p < 0.0001).

We next characterized the immune cell composition of the upper and lower lung compartments following IAV infection. IAV infection induced macrophage, monocyte, pDC, NKT cell, eosinophil, T cell and B cell infiltration in the nasal tissue of WT mice (Fig. 4). In TLR7 KO mice, there was a suppression of Ly6C^lo^ and Ly6C^hi^ monocytes, pDCs and B cells, while NK cell numbers were higher compared to WT mice. TLR7 deficiency did not alter the frequency of macrophages, eosinophils, NKT cells or T cells in response to IAV infection in this tissue. In the BAL, infection led to an increase in the frequency of macrophages, monocytes, neutrophils, DCs, NK cells, NKT cells, eosinophils, T cells and B cells in WT mice (Fig. 5). However, in TLR7 KO mice, the immune response was blunted for all cell types except pDCs and NK cells. TLR7 KO mice also displayed significantly more pDCs in the airways. Similarly, in the lung tissue, infection led to an increase in the frequency of macrophages, monocytes, neutrophils, classic DCs, NK cells, NKT cells, eosinophils, T cells, and B cells in WT mice (Fig. 6). In TLR7 KO mice, infiltration of all cell types was significantly reduced, except for pDCs, which increased in number, and eosinophils, which exhibited a response similar to that of WT mice.

**Fig. 7:**
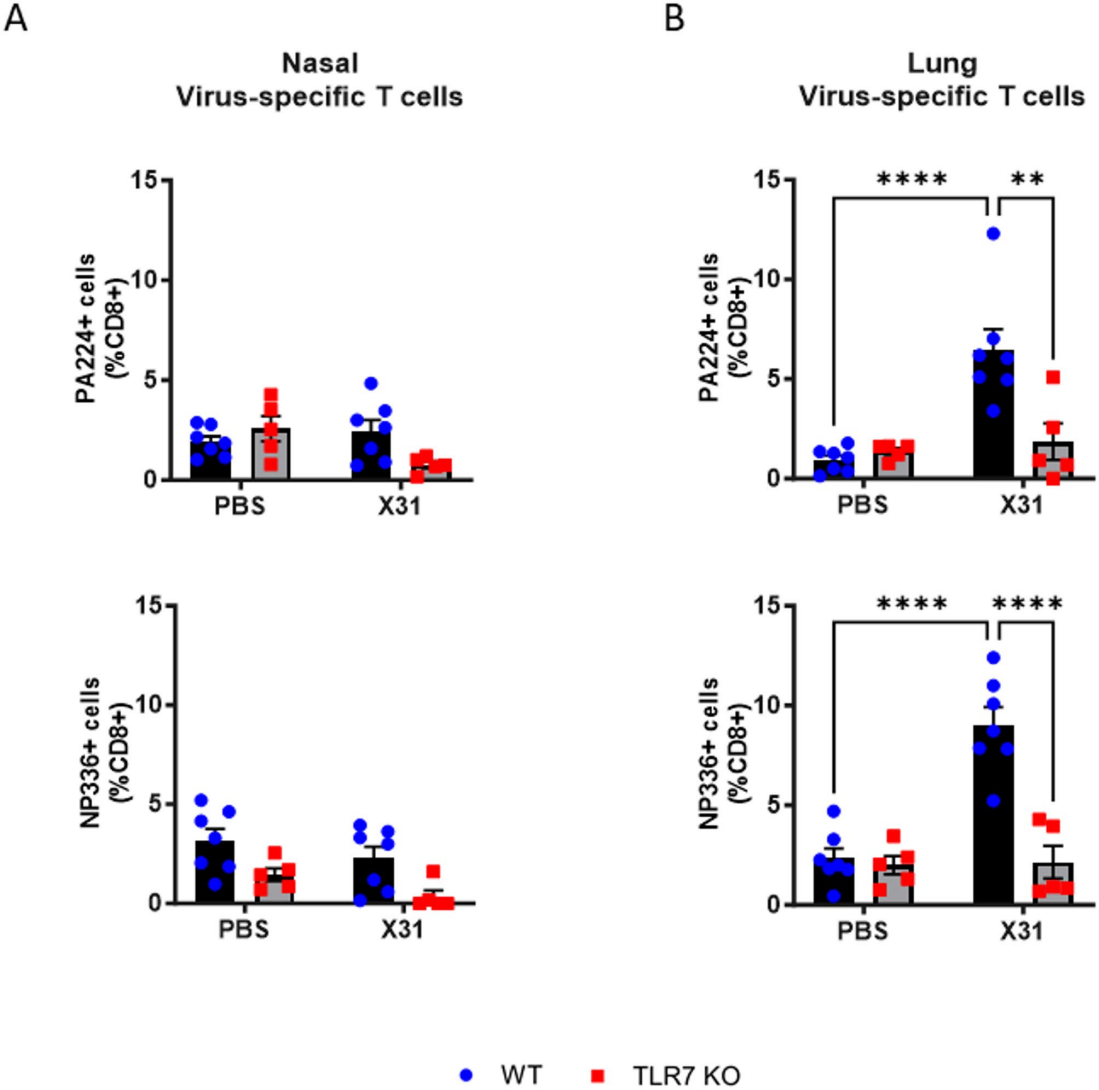
TLR7 promotes virus-specific T cells in the lungs but not the nasal tissue following X31 infection. WT C57Bl/6 or TLR7 KO mice were infected with Hk-X31 (10^4^ PFUs) or PBS (control) and cells isolated from the (**A**) nasal or (**B**) lung tissues after 6 days. Virus-specific cytotoxic T cells expressing the epitopes PA_224–233_ and NP_336 − 374_ were gated from CD8 + cells. Data is expressed as mean ± SEM, *n* = 5–7 biological replicates. Statistical analysis was conducted using two-way ANOVA test followed by Tukey’s post hoc test for multiple comparisons (**p < 0.01, ****p < 0.0001).

We also assessed the frequency of CD8 + T cells expressing the viral epitopes PA_224–233_ and NP_336 –374_, as a measure of T cell-mediated immunity. Virus-specific CD8 + T cells were not detected in the nasal tissue (Fig. 7A) but were present in the lungs (Fig. 7B). These T cells were observed only in WT mice, suggesting that a virus-specific T cell response is absent in the URT but occurs in the LRT, at least during the acute phase, and this response relies on TLR7.

These findings indicate that TLR7 is essential for a robust innate and adaptive immune response in the LRT following IAV infection. In the URT, TLR7 primarily drives a strong monocyte, pDC and B cell response, but does not significantly affect the macrophage or T cell responses.

### TLR7 deficiency reduces inflammation in the LRT but increases it in the URT following IAV infection

**Fig. 8:**
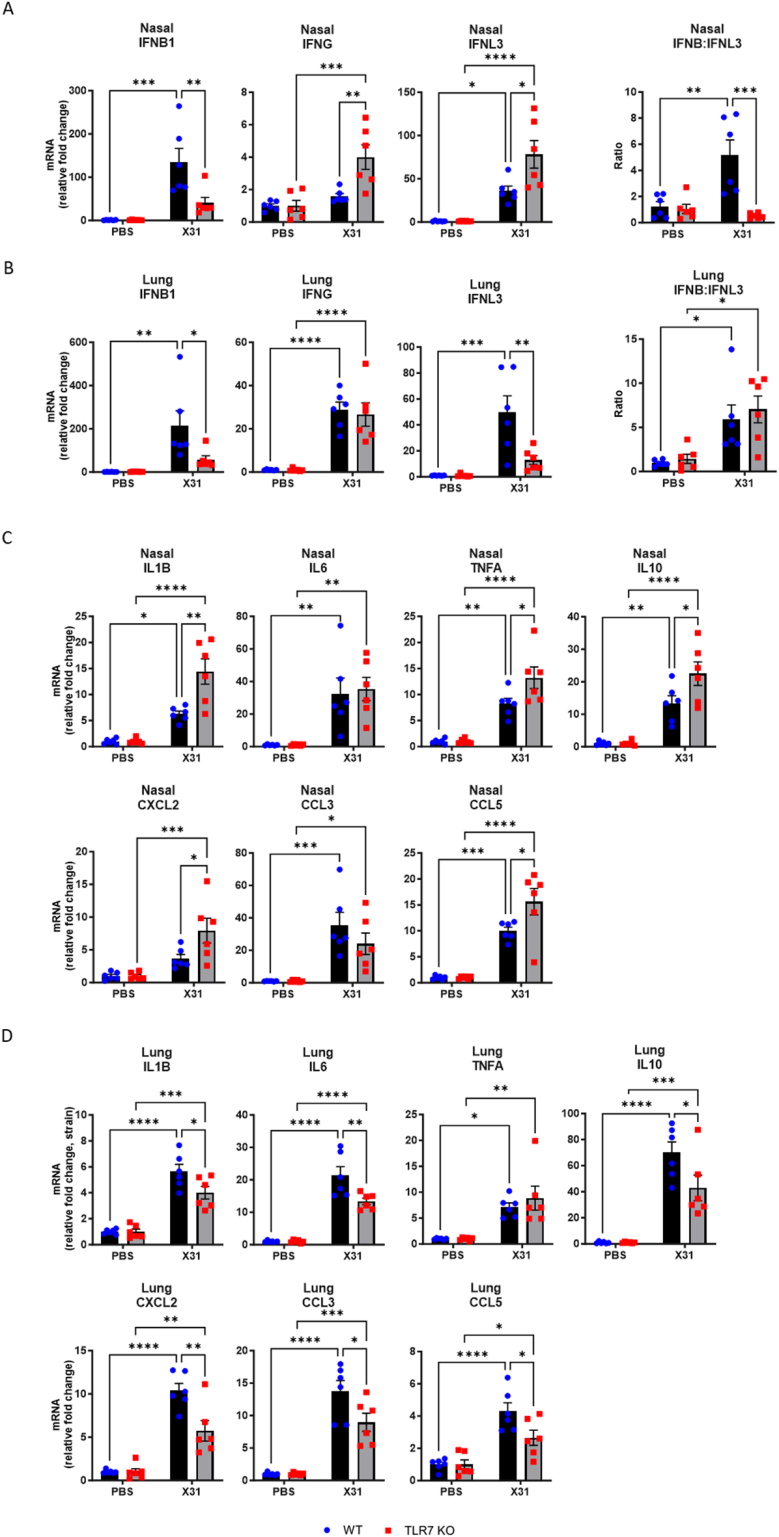
TLR7 KO mice exhibit increased proinflammatory gene expression in the URT following X31 infection. WT C57Bl/6 or TLR7 KO mice were infected with Hk-X31 (10^4^ PFUs) or PBS (control). Nasal and lung tissues were isolated after 6 days and gene expression analysed by RT-qPCR. (**A**, **B**) Type I, II and III IFNs or (**C**, **D**) proinflammatory genes are presented relative to RPS18 housekeeping as a fold change above PBS controls. Data is expressed as mean ± SEM, *n* = 3 technical replicates from 6 biological replicates for each strain. Statistical analysis was conducted using two-way ANOVA test followed by Tukey’s post hoc test for multiple comparisons (*p < 0.05, **p < 0.01, ***p < 0.001, ****p < 0.0001).

To gain further insight into the immune pathways affected by TLR7 deficiency in the URT and LRT, we assessed the expression of IFNs, proinflammatory cytokines, and chemokines in nasal and lung tissues. Infected WT mice showed a clear *IFNB1* and *IFNL3* response in the nasal tissue. In contrast, TLR7 KO mice displayed significantly higher *IFNG* and *IFNL3* levels, while the *IFNB1* response was blunted (Fig. 8A). Analysis of the type I to type III IFN ratio, measured as *IFNB1*: *IFNL3*, indicated a dominant type I IFN response in the nasal tissue of WT mice, which was absent in TLR7 KO mice. All IFNs were upregulated in the lungs of infected WT mice (Fig. 8B). However, the upregulation of *IFNB1* and *IFNL3* was significantly reduced in the lungs of TLR7 KO mice, and IFN type I: III ratios indicated a dominant type I IFN response in both genotypes. Various inflammatory cytokines and chemokines were also upregulated in the nasal tissue of both genotypes following infection (Fig. 8C). *IL1B*, *TNFA*, *IL10*,* CXCL2* and *CCL5* expression was significantly higher in TLR7 KO nasal tissue compared to WT. In lung tissue, *IL1B*, *IL6*, *IL10*,* CXCL2*, *CCL3* and *CCL5* levels also rose in both genotypes following infection, although these markers were significantly lower in TLR7 KO mice (Fig. 8D).

These analyses indicate that although viral titres were not different between genotypes in both tissues, TLR7 deficiency suppressed the type I IFN response, and enhanced the expression of type II /III IFN and specific proinflammatory markers in the URT. In contrast, TLR7 deficiency reduced the expression of inflammatory markers in the LRT.

## Discussion

This study aimed to characterize the immune response mediated by TLR7 in the URT and LRT using a moderately pathogenic model of IAV. TLR7 KO mice exhibited increased levels of type II/III IFNs and proinflammatory cytokines in the URT, while showing a decrease in overall inflammatory responses in the LRT. This pattern was associated with significant alterations in both innate and adaptive immune cell populations in the airways and lungs of TLR7 KO mice. Despite these immune alterations, the responses of macrophages, NKT cells, eosinophils, and T cells remained largely intact in the nasal tissue. These findings suggest that TLR7 plays a crucial role in driving immune responses in the LRT, but its role in the URT is more limited. Additionally, the expression and stimulatory capacity of TLR7 were highest in cells of the LRT, reinforcing its predominant function in driving LRT inflammation. The reduced body weight loss observed in TLR7 KO mice highlights TLR7 as a key driver of LRT disease during IAV infection.

The role of TLR7 in the URT and LRT was clearly distinct in terms of immune response. Our study mapped TLR7 expression in various regions of the URT and LRT, providing valuable insight into the spatial distribution of immune responses during IAV infection. The findings reveal that TLR7-driven inflammatory responses are predominantly localized to the LRT, where macrophages and monocytes are the primary cell types expressing TLR7. Hyperactivation of macrophages and monocytes, in particular, in the LRT has been strongly associated with poor clinical outcomes during IAV infection, highlighting the potentially detrimental role of TLR7 signalling in exacerbating lung pathology^16,29,30^. In contrast, while TLR7 was also detected in the nasal tissue, its expression was markedly lower compared to the LRT. Notably, TLR7 expression in the nasal tissue was largely confined to immune cells, such as macrophages and NK cells. These data suggest that the contribution of TLR7 to the URT inflammatory response has been underestimated in current literature, likely due to its relatively low expression in epithelial cells^26^, which are commonly used to model viral infection. Our study, therefore, offers a more comprehensive view of TLR7 responses to IAV infection in the nasal compartment by also considering both resident and infiltrating immune cells.

This investigation into TLR7 expression in nasal tissues highlights the importance of the immune microenvironment in the URT, including specialised structures like the nasal-associated lymphoid tissue (NALT), which plays a crucial role in initiating mucosal immune responses to pathogens. The nasal immune microenvironment, comprising macrophages, DCs, and NK cells, functions to survey the local area and initiate mucosal immune responses to combat invading pathogens^31^. Many of the TLR7-expressing cells we observed are contained within the NALT and this specialized lymphoid structure is rich in B cells, T cells, macrophages, and DCs^32,33^. NK cells, which are recruited to the NALT following intranasal immunization, have also been shown to be activated by TLR7 during IAV infection^34,35^. Moreover, the NALT is known to play a pivotal role in the generation of CD8 + T cell memory responses following IAV infection, with the priming of naïve CD8 + T cells occurring at the draining cervical lymph nodes^36^. This may help explain why we did not detect IAV-specific T cells in the nasal tissue, as these cells are likely primed and recruited to the lung, where the bulk of the immune response and viral clearance occur. Future experiments examining later timepoints will be needed to validate this hypothesis.

After mapping TLR7 expression and immune cell contributions in the URT, it was essential to explore how TLR7 deficiency affected the immune responses in the airways and lungs, especially during IAV infection. The effects of TLR7 deficiency were most pronounced in the airways and lungs, where both innate and adaptive immune responses were significantly altered. This resulted in a marked reduction in proinflammatory gene expression in the lungs of TLR7 KO mice compared to WT mice, suggesting that TLR7 is important for driving robust inflammatory responses in the LRT. In contrast, TLR7 deficiency in the nasal tissue impaired responses from monocytes, pDCs, and B cells, while macrophage, NK cell, eosinophil, and T cell responses remained largely intact. Interestingly, despite these immune deficiencies, TLR7 KO mice exhibited higher expression of proinflammatory markers, as well as elevated levels of TLRs and RLRs, in the nasal tissue compared to WT mice. This suggests a compensatory upregulation of alternative immune pathways that might help mitigate the effects of TLR7 deficiency in the absence of overt pathology, thereby reducing disease severity that may be driven by TLR7-mediated inflammation. Indeed, intact antiviral and some T cell responses to IAV infection can occur via IL-1R or MAVS signalling in the absence of TLR7^37–39^. These compensatory responses may be more pronounced in the URT, given the higher expression of those components in this region, possibly resulting in the observed intact macrophage, NKT and T cell responses in the nasal tissue. Additionally, increased levels of *IFNG*, which likely reflects the heightened presence of NK cells in the nasal tissue, were observed. Anti-inflammatory *IL10* expression mirrored the levels of proinflammatory cytokines, both in the URT and LRT, potentially indicating the host’s attempt to resolve inflammation and limit tissue damage^40^. Overall, TLR7 KO mice showed a higher degree of inflammation in the URT and reduced inflammation in the LRT compared to WT mice. Despite these differences in inflammatory profiles, viral load in both nasal and lung tissues was similar between TLR7 KO and WT mice, suggesting that TLR7 does not significantly affect viral replication at these sites, at least at the time point studied. This finding highlights the complex role of TLR7 in modulating the immune response, rather than directly influencing viral load.

This duality in immune responses suggests that TLR7 acts pivotally in the inflammatory response to IAV infection, particularly by regulating the transition of inflammation from the URT to the LRT. In the absence of TLR7, inflammation remains sustained in the URT, potentially preventing excessive inflammatory damage in the lungs. This hypothesis is further supported by our observation that TLR7 KO mice exhibited less body weight loss during infection, suggesting a less severe disease course compared to WT mice. Disease severity is often attributed to changes in lung histopathology. Further studies that comprehensively assess histological alterations across multiple regions of the respiratory tract, and examine how these changes correlate with inflammatory markers in these tissues, will provide additional insight into how TLR7-mediated inflammation contributes to disease progression. It is important to recognize, however, that impaired immune memory to IAV infection could be a long-term consequence of TLR7 deficiency^39,41^. Although previous research has linked enhanced neutrophilic and monocytic inflammatory signatures in nasal samples to poorer IAV outcomes—reflecting the extensive inflammation observed in the LRT^6,17^—our data indicate that the higher inflammatory responses in the URT at day 6 post-infection might actually be protective, especially when accompanied by reduced inflammation in the LRT. In addition, localized inflammation in the URT, such as that caused by bacterial colonization in the nasal mucosa, has been shown to mitigate LRT disease during subsequent IAV infections^42,43^, further suggesting that localized inflammation in the URT can be protective. Furthermore, we found that the monocyte response, which can drive lung damage during IAV infection^16,29^, was impaired in both the URT and LRT of TLR7 KO mice, suggesting that these mice may be protected from monocyte-driven pathology. Further work is needed to fully understand the role of TLR7 in monocyte-mediated IAV disease and its broader implications for immune regulation.

A potential mechanism underlying the TLR7-dependent propagation of inflammation from the URT to the LRT may involve the differential contributions of type I and type III IFN signalling during IAV infection, specifically in the URT. Galani et al.^9^ demonstrated that, upon IAV infection, IFN-λ is initially produced at the epithelial barrier, followed by the production of type I IFN, which drives proinflammatory cytokine production and immune cell recruitment. Importantly, a higher viral inoculum was found to trigger a more rapid type I IFN response, leading to earlier inflammation and more severe disease outcomes. Moreover, Davidson et al.^44^ showed that therapeutic administration of IFN-λ during active IAV infection reduced viral replication in the airway epithelia and conferred protection, while IFNα stimulated inflammation in the lungs and exacerbated LRT disease. Similar protective effects of intranasal IFN-λ have also been observed in mice infected with SARS-CoV-2^45^. These findings underscore that type III IFNs exert less immunostimulatory effect on immune cells compared to type I IFNs during infection, suggesting that an early dominance of type I IFN responses may drive more extensive inflammation in the LRT. This could be due to the broader expression of the IFNAR1 across many cell types, whereas the IFN-λ receptor (IFNLR1) is primarily expressed on epithelial cells. As a result, type III IFNs exert their effects more specifically at mucosal surfaces, providing antiviral protection without driving excessive proinflammatory responses^46^. In terms of the IFN response in the lungs, we found lower type I and type III IFNs in TLR7 KO mice following IAV infection. We hypothesize that this reduced response specifically in the lungs may be protective, as sustained type I IFN signalling in the LRT can impair lung epithelial regeneration and repair during the later stages of infection, contributing to chronic inflammation and fibrosis^47^.

In contrast, in the URT, TLR7 deficiency had a more specific effect, primarily reducing *IFNB1* production while enhancing *IFNL3* levels. This shift resulted in a more balanced type I/III IFN response in the nasal tissue of TLR7 KO mice, compared to a dominant type I IFN response in WT mice. We hypothesize that this altered IFN response in TLR7 KO mice leads to reduced recruitment and activation of inflammatory cells to the LRT without altering viral load, thus protecting these mice from severe LRT disease. Although further work is needed to mechanistically validate this hypothesis, we propose that TLR7-dependent production of type I and III IFNs by specific cell types, and its temporal dynamics, plays a crucial role in this process. Type III IFNs are primarily produced by the respiratory epithelium during IAV infection, with pDCs also contributing to this production^9,48^. We speculate the primary source of IFN-λ in the URT of TLR7-deficient mice to derive primarily from epithelial cells, possibly through RIG-I/MDA5 recognition^49^ since TLR7 expression is minimal in these cells and RLR expression was found to be elevated in the nasal tissue after infection. In contrast, pDCs, which typically express TLR7, would have a diminished capacity to produce type I and III IFNs to IAV infection in these mice^50^. This would lead to an antiviral response predominately regulated by epithelial-derived IFN-λ, without the additional proinflammatory effects of type I IFN. We therefore propose that TLR7 exacerbates IAV disease by skewing the immune response in favour of type I IFN, potentially sustaining IFN production for longer than necessary, which in turn promotes sustained downstream LRT inflammation. Supporting this, PBMCs treated with the TLR7/8 agonist R848 secreted more IFN-α than IFN-λ, highlighting the capacity of TLR7 to bias the immune response toward type I IFN^51^. Furthermore, SARS-CoV-2 infection of pDCs promoted type I IFN production via TLR7, and this led to transcriptional and epigenetic changes in lung macrophages that enhanced their proinflammatory phenotype^22^. Thus, these TLR7-dependent events in the URT, along with the production of IFNs, may influence the inflammatory status of cells in the LRT, contributing to the overall immune response and potentially exacerbating disease outcomes.

In summary, this study highlights the pivotal role of TLR7 in shaping the immune response during IAV infection, providing evidence that TLR7 mediates the dissemination of the inflammatory response from the URT to the LRT. We utilized an acute IAV model of Hk-X31 infection thereby enabling us to assess concordant immune responses in both the URT and LRT. Future work assessing the temporal progression of immune alterations in across multiple compartments of the respiratory tract will provide a more comprehensive understanding of the immune dynamics driven by TLR7 during IAV infection. Furthermore, characterising the dissemination of inflammation from the URT to LRT following infection with more pathogenic strains of IAV will provide important insights into the broad role of TLR7. Previous studies have shown that either activating TLR7 via IMQ prior to IAV infection or inhibiting it with IRS661 during established infection reduces LRT pathology^21,52^. These treatments were administered intranasally, implying that TLR7 within cells of both the URT and LRT were targeted, although the immune responses in the URT were not specifically addressed. Building on these findings, our results imply that early intranasal administration of TLR7 antagonists could be a more effective approach in IAV treatment. Such a strategy may have two key mechanistic outcomes to limit LRT inflammation: (1) it could reduce the immunostimulatory effects of type I IFNs, thereby suppressing the indirect activation and recruitment of downstream inflammatory cells, and (2) it could dampen the direct activation of TLR7-expressing immune cells. By limiting excessive inflammation in the LRT, this approach could alleviate IAV disease severity. Therefore, targeting TLR7 may be a therapeutic strategy to improve outcomes in severe IAV infections by modulating pathogenic responses in both the upper and lower respiratory tracts.

## Methods

### Animal ethics

All experimental protocols involving animals were approved by the RMIT University Animal Ethics Committee (Ethics number 23328, 24336) and conducted in compliance with the guidelines of the National Health and Medical Research Council of Australia on animal experimentation. All methods and authors complied with ARRIVE guidelines.

### Mice and IAV infection

Female C57BL/6J mice were obtained from the Animal Resources Centre (Western Australia, Australia). Homozygous TLR7 knockout mice (B6.129S1-Tlr7tm1Flv/J) were obtained from The Jackson Laboratory (Maine, USA) and bred in-house at the RMIT University animal research facility (Bundoora, Australia). Mice were housed in a 12 h light/12 h dark cycle with food and water. Mice were 8–12 weeks old at the commencement of experiment. Female mice were used in this study as they generally exhibit stronger innate immune responses to IAV infection.

8-12-week-old mice were anaesthetized by isoflurane inhalation and inoculated intranasally with PBS (to serve as mock-infected controls) or 10^4^ plaque forming units (PFUs) of mouse-adapted HKx31 virus (H3N2 strain) in a 35 µL volume. Mice were weighed and monitored daily. Mice were euthanized by injection (i.p) of a mixture of sodium pentobarbital (325 mg/ml) and lignocaine (20 mg/ml) at the experimental endpoint. The 6 day post infection final timepoint was chosen as it ensures detectable inflammation and viral presence in both the URT and LRT compartments, along with significant body weight loss to provide a clear indication of disease morbidity.

### Recovery of immune cells

Cells from the nasal tissue (nasal cavity and nasal turbinates) and lung tissues were prepared by brief mincing with scissors then enzymatically digested in 1% Liberase (Sigma) for 30 (nasal) or 45 (lung) min at 37 °C shaking at 700 rpm. Single cell suspensions were prepared by straining homogenized tissue through a 40 μm strainer, and the red blood cells lysed with ACK lysis buffer. Bronchoalveolar lavage (BAL) was performed to extract cells from the lower airways. This was achieved by inserting a sheathed 21-Gauge needle into a small incision on the trachea and the lungs lavaged with 300–400 µL aliquots of PBS repeatedly with gentle massaging of the chest until a volume of 1 mL was collected. All cells were maintained in PBS + 2.5% FBS until analysis.

### Flow cytometry

Whole lung (finely minced using scissors) or nasal tissue was enzymatically digested using 1% Liberase (Sigma) for 30 (nasal) or 45 (lung) min at 37 °C shaking at 700 rpm. Tissues were homogenized then single cell suspensions prepared by straining through a 40 μm strainer. BAL cells were used without any further handling. After lysing the red blood cells with ACK lysis buffer, cells were stained with cocktail mixtures of fluorescent-labelled anti-mouse antibodies diluted in FACS buffer (PBS + 2.5% FBS) for 30 min on ice. The following Biolegend antibodies were used (unless stated otherwise): CD45-Alexa Fluor 700 (clone 30-F11), CD45-BV605 (clone 30-F11), CD3-APC (clone 17A2; eBioscience), CD4-BV605 (clone RM4-5), CD8a-PerCP (clone 53 − 6.7), NK1.1-FITC (clone PK136), CD11b-BV421 (clone M1/70), CD11b-APC-Cy7 (clone M1/70), CD11c-PE-Cy7 (clone N418; eBioscience), PDCA-1-PE (clone JF05-01C2.4.1; Miltenyi Biotec), MHC-II-APC (clone M5/114.15.2), Ly6C-PerCP (clone HK1.4), Ly6G-APC-Cy7 (clone 1A8), Siglet-F-PE (clone 1RNM44N; ThermoFisher), F4/80-PE (clone BM8; eBiocience) and B220-FITC (clone RA3-6B2). Tetramer staining of virus-specific CD8 + T cells was performed using PE or APC-conjugated peptides D^b^NP_366_ and D^b^PA_224_ that were synthesised by the Innate Immunity and Anti-Viral Immunity Laboratory in the Department of Microbiology and Immunology, University of Melbourne. In some experiments, cells were fixed and permeabilized using the eBioscience Foxp3 / Transcription Factor Staining Buffer Set (ThermoFisher) for intracellular staining with rabbit anti mouse-TLR7 (clone NBP224906; Novus Biologicals). After 30 min of intracellular staining, cells were stained with secondary goat anti-rabbit IgG-AF594 (Abcam) for a further 30 min. CD16/32 (clone 2.4G2) and LIVE/DEAD Fixable Aqua Dead Cell Stain Kit (Invitrogen) were contained within each antibody cocktail mixture to block of Fc-mediated adherence of the antibodies and to exclude dead cells, respectively. Samples were processed on a BD LSRFortessa X-20 flow cytometry analyzer with DIVA software (Becton Dickinson Bioscience, USA) and data analyzed using FlowJo software (Tree Star, Inc.). The data is represented as a percentage of live cells. The representative gating strategy is shown in Supplementary Fig. 2.

### Ex vivo treatment of tissue

One hundred thousand cells from nasal, BAL or lung tissues were seeded in each well of a 96-well plate containing complete DMEM (10% FBS, 1% penicillin-streptomycin). Cells were then exposed to 1 µg/ml of the TLR7 agonist imiquimod (IMQ; Invivogen) or left untreated for 18 h. This concentration was used as higher concentrations of IMQ may activate inflammatory responses independent of TLR7 ^53^. Following treatment, media was aspirated and cells directly lysed for RNA extraction and subsequent gene expression analysis.

### RNA extraction and RT-qPCR

Total RNA was extracted from nasal or lung homogenates using the RNeasy Mini kit (Qiagen, USA), and RNA quality validated using a Nanodrop 2000 spectrophotometer (ThermoFisher, CA, USA). 1–2 µg of RNA was converted to cDNA using the High-Capacity cDNA Reverse Transcription Kit (Applied Biosystems, CA, USA) according to manufacturer’s instructions. Amplification of target genes were evaluated using pre-designed TaqMan primers (Life Technologies, CA, USA) and the TaqMan Fast Advanced Master Mix (ThermoFisher). A custom designed TaqMan primer containing the oligonucleotide sequences 5′-CGGTCCAAATTCCTGCTGA-3′, 5′-CATTGGGTTCCTTCCATCCA-3′ was used to amplify the influenza A polymerase gene. Amplification was performed using a QuantStudio 7 Flex Real-Time PCR system (Thermofisher) according to the following program: 50 °C for 2 min, 95 °C for 2 min, then 40 cycles of 95 °C for 1 s and 60 °C for 20 s. Gene quantitation was performed in triplicate and analysed as a fold change relative to uninfected controls using the comparative Ct method normalised against the housekeeping gene RPS18. Basal expression is presented as 2^−ΔCt^ indicating the difference in threshold cycle between the RPS18 and the investigated PRR gene.

### Statistics and reproducibility

Data are presented as mean ± SEM from at least two independent experiments. Statistical comparisons were made using two-way ANOVA followed by Tukey’s post-hoc test for multiple comparisons. A *p* value of less than 0.05 was considered statistically significant. All statistical analyses were performed using GraphPad Prism (Version 10, USA). Statistical details of experiments including n numbers can be found in figure legends.

## Electronic supplementary material

Below is the link to the electronic supplementary material.

Supplementary Material 1

## Data Availability

All data generated or analyzed during this study are included in this published article (and its Supplementary Information files) and are available from the corresponding author on reasonable request.
