# Supplementary material for: TLR7 deficiency enhances inflammation in the URT but reduces LRT immunity following influenza A infection
Source: Sci Rep. 2025 May 29;15:18918. doi: 10.1038/s41598-025-04154-6 (PMC12123008; doi:10.1038/s41598-025-04154-6)
Supplement: Supplementary file 1 — Supplementary Material 1 [file 41598_2025_4154_MOESM1_ESM.pdf]

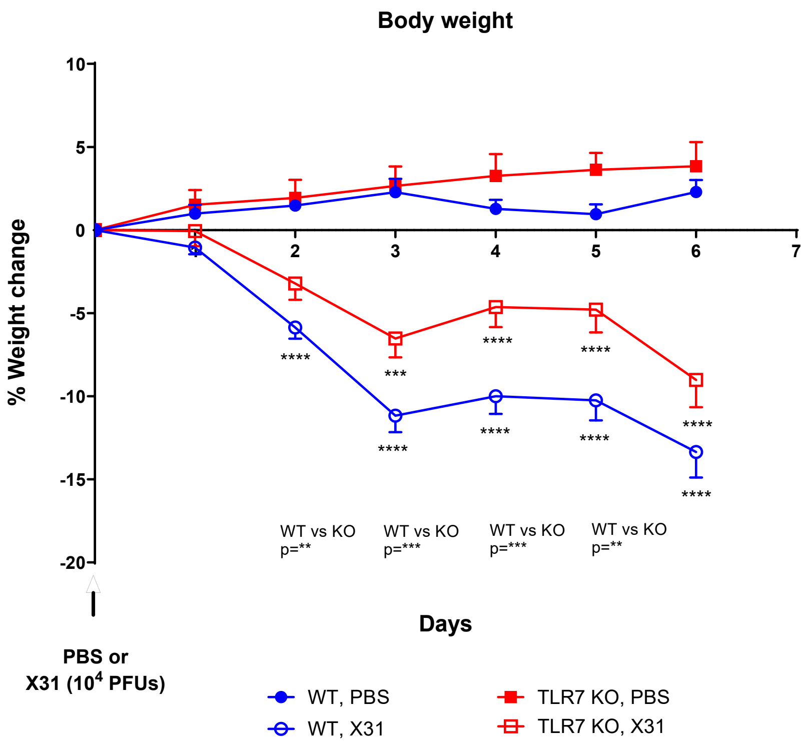

Supplementary Figure 1. TLR7 KO mice exhibit reduced acute body weight loss following X31 infection. WT C57Bl/6 or TLR7 KO mice were infected with Hk-X31 (10^4 PFUs) or PBS (control). Body weights were recorded daily for 6 days and presented as % weight change from day of infection. Data is expressed as mean ± SEM, n = 9-13. Statistical analysis was conducted using two-way ANOVA test followed by Tukey's post hoc test for multiple comparisons (**$p < 0.01$, ***$p < 0.001$, ****$p < 0.0001$).

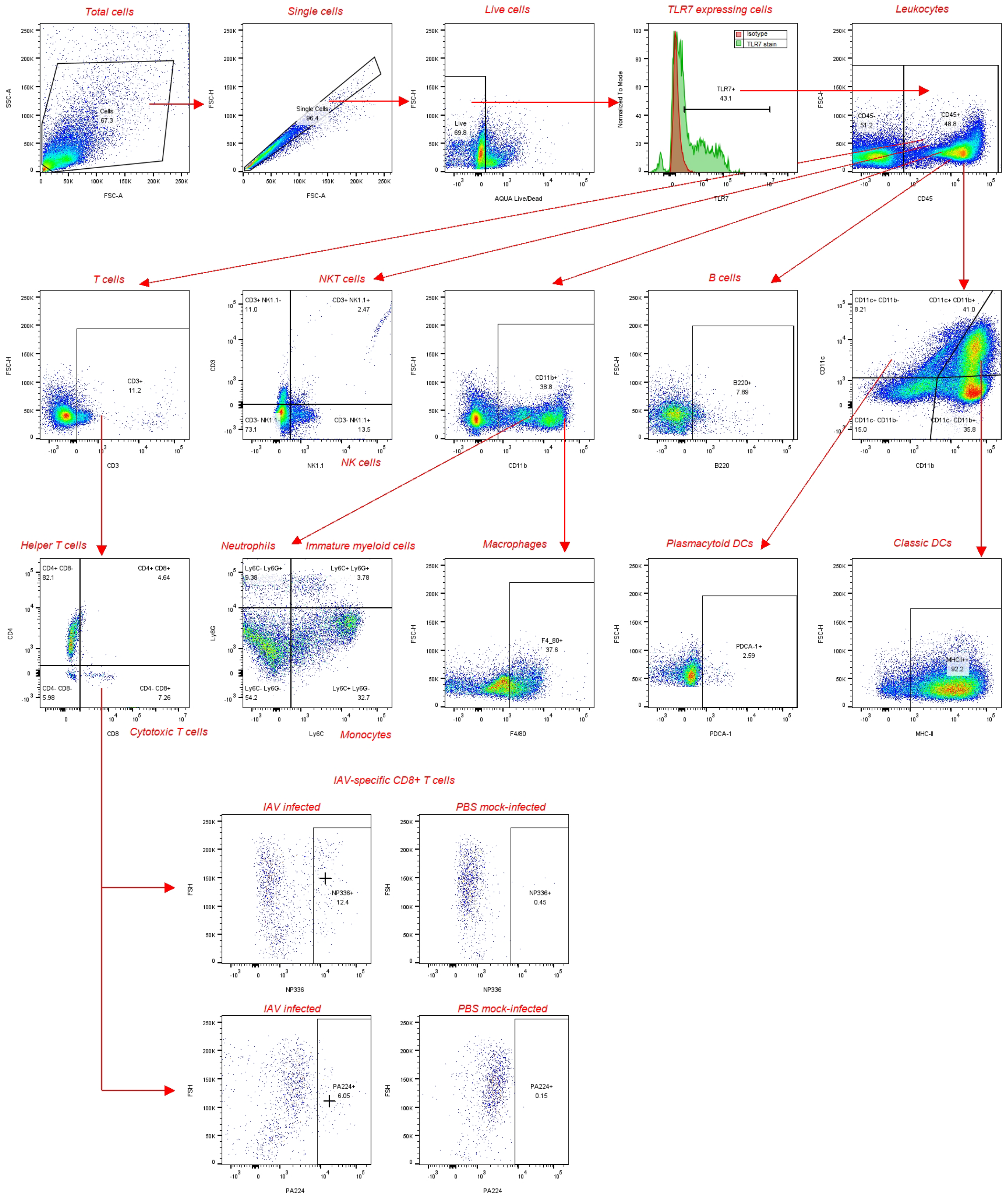

Supplementary Figure 2. Flow cytometry gating strategy. For analysis that did not consider TLR7 expression, leukocytes were gated from live cell populations.
